# Children who idiopathically toe-walk have greater plantarflexor effective mechanical advantage compared to typically developing children

**DOI:** 10.1007/s00421-022-04913-7

**Published:** 2022-03-16

**Authors:** Carla Harkness-Armstrong, Constantinos Maganaris, Roger Walton, David M. Wright, Alfie Bass, Vasilios Baltzoloulos, Thomas D. O’Brien

**Affiliations:** 1 Research Centre for Musculoskeletal Science and Sports Medicine, Manchester Metropolitan University, Manchester, UK; 2 Research Institute for Sport and Exercise Sciences, Liverpool John Moores University, Tom Reilly Building, Byrom Street, Liverpool, L3 3AF UK; 3 Alder Hey Children’s NHS Foundation Trust, Liverpool, UK

**Keywords:** Mechanical advantage, Moment arm, Achilles tendon, Equinus, Idiopathic toe-walking

## Abstract

**Purpose:**

The effective mechanical advantage (EMA) of the plantarflexor muscles is important for gait function and is likely different from typical in equinus gait. However, this has never been quantified for children who idiopathically toe-walk (ITW), despite being routinely altered through clinical intervention.

**Methods:**

This study quantified the Achilles tendon and ground reaction force (GRF) moment arms, and the plantarflexor EMA of 5 children who ITW and 14 typically developing (TD) children, whilst walking on an instrumented treadmill.

**Results:**

There was no difference in the Achilles tendon moment arm length throughout stance between groups (*p* > 0.05). Children who ITW had a significantly greater GRF moment arm length in early stance (20–24% *p* = 0.001), but a significantly shorter GRF moment arm length during propulsion (68–74% of stance; *p* = 0.013) than TD children. Therefore, children who ITW had a greater plantarflexor EMA than TD children when active plantarflexion moments were being generated (60–70% of stance; *p* = 0.007). Consequently, it was estimated that children who ITW required 30% less plantarflexor muscle force for propulsion.

**Conclusion:**

Clinical decision making should fully consider that interventions which aim to restore a typical heel-toe gait pattern risk compromising this advantageous leverage and thus, may increase the strength requirements for gait.

**Supplementary Information:**

The online version contains supplementary material available at 10.1007/s00421-022-04913-7.

## Introduction

Efficient locomotion requires the generation of adequate contractile muscle force to overcome the effects of external forces acting on the body. The required magnitude of contractile force is partly determined by the leverage about which the muscle and ground reaction forces (GRF) act on the skeleton. For the plantarflexors, this leverage, known as effective mechanical advantage (EMA), is quantified as the ratio between moment arms of the internal Achilles tendon force and external GRF (Biewener et al. 2004). Moment arms are defined as the shortest perpendicular distance between the line of action of force and the axis of rotation. Both moment arms can be altered by anatomical variations and/or kinematic changes, which in turn greatly influence the muscle force required to generate adequate joint moments (Lee and Piazza 2009).

Children who idiopathically toe-walk (ITW) walk in equinus despite no diagnosed orthopaedic or neurological disorder (Sala et al. 1999). This altered gait pattern likely affects the plantarflexor EMA. In simulated toe-walking (Kerrigan et al. 2000) and in simple two-dimensional models of cerebral palsy (CP) (Hampton et al. 2003), equinus gait shortens the external GRF moment arm length, which consequently improves plantarflexor EMA and reduces the plantarflexor muscle force required. Therefore, equinus gait has been suggested to be, in part, a compensatory mechanism for plantarflexor weakness (Hampton et al. 2003). However, children who ITW do not present with plantarflexor weakness and indeed, were shown to operate close to optimal joint angle during gait (Harkness-Armstrong et al. 2021).

Reducing the GRF moment arm length may therefore be a mechanism to compensate for anatomical changes in the Achilles tendon moment arm. In children (Kalkman et al. 2017) and young adults (Gallinger et al. 2020) with CP the Achilles tendon moment arm has been found to be shorter than typical, which has been attributed to chronic atypical loading of the ankle joint. Consequently, it has been suggested that for these individuals, actively reducing the GRF moment arm length may restore the ratio of EMA back to typical, rather than improve it (Kalkman et al. 2017). Children who ITW experience this same chronic atypical loading of the ankle joint, therefore these children might undergo similar skeletal alterations as children with CP, resulting in similar alterations in plantarflexor leverage. Therefore, it is essential to understand whether an altered musculoskeletal leverage may be impacting the muscle force requirements and/or contributing to movement impairments in children who ITW, to ensure that appropriate clinical interventions can be provided.

Treatments for equinus gait in children who ITW aim to restore dorsiflexion range of motion and a typical heel-toe gait pattern, as if untreated, it can lead to muscle contracture (Solan et al. 2010), fixed deformity (Dietz and Khunsree 2012) and worsening of symptoms (Sobel et al. 1997). Such interventions include serial casting (Brouwer et al. 2000; Fox et al. 2006), botulinum toxin-A injections (Brunt et al. 2004; Satila et al. 2016), a combination of the two (Engström et al. 2013; Gormley et al. 1997), or orthopaedic surgery if a contracture is already present (Hemo et al. 2006; Jahn et al. 2009; McMulkin et al. 2006).

In children with CP, similar clinical interventions can cause plantarflexor weakness (Gage et al. 2009; Orendurff et al. 2002). This may be because these treatments increase the GRF moment arm length, without full consideration of the impact on ankle moment requirements for locomotion. It is likely that these treatments negatively alter the plantarflexor EMA and thus, increase the plantarflexor muscle force required for gait. Moreover, we have recently shown that children who ITW present with substantial alterations in the gastrocnemius muscle functional properties, with the optimum joint angle in more plantarflexed positions than typical, but well aligned with the active range of motion during gait (Harkness-Armstrong et al. 2021). Consequently, treatments may, in the short-term, shift children who ITW away from their optimum joint angle. This problem would then be compounded if EMA is reduced, with the effect being that the child would be weakened while they are simultaneously required to produce greater muscle forces post-intervention. This may explain why current clinical interventions have poor medium to long-term outcomes for children who ITW, with high rates of recurrence (Dietz and Khunsree 2012; van Kuijk et al. 2014). However, the effect of such interventions on plantarflexor EMA has not been measured. Moreover, it is also important to consider that although the primary aim of such interventions is to improve the biomechanics of children who ITW, clinicians must also consider other factors such as foot pain (Fox et al. 2006) and gait stability (Soangra et al. 2021).

Despite the evidence indicating that plantarflexor leverage is important for gait function and is likely altered through clinical intervention, it has never been quantified for children who ITW. Whilst it is rather rare for ITW to persist (not including recurrence) beyond early childhood (Engström et al. 2012), the gait pathology does continue in a small number of children who often require clinical treatments. Therefore, although this makes children who ITW a difficult population to recruit experimentally for cross-sectional research, it is important to understand whether the plantarflexor EMA of children who ITW differs from children who walk with a typical heel-toe gait pattern, and whether this altered leverage forms part of the pathology or is a compensation strategy. This knowledge is necessary to fully inform clinical decision-making. Recently, we developed a novel methodology that can overcome the anatomical challenges of measuring plantarflexor EMA in equinus gait, including accounting for Achilles tendon curvature in extreme plantarflexed positions (Harkness-Armstrong et al. 2020). Thus, the aim of this study was to use this method to determine the plantarflexor EMA during gait in TD children and children who ITW. We hypothesised that children who ITW would present with a similar plantarflexor EMA as TD children, similarly to the proposed effect in children with CP, despite the altered gait pattern.

## Method

### Participants

Five children who bilaterally ITW (male *n* = 2; female *n* = 3; age 8 ± 2yrs.; height 1.38 ± 0.15 m; body mass 45.2 ± 26.7 kg) and 14 TD children (male *n* = 5; female *n* = 9; age 10 ± 2yrs.; height 1.39 ± 0.11 m; body mass 37.8 ± 17.5 kg) were recruited for this study. Children who ITW were recruited from outpatient lists at a hospital gait laboratory and orthopaedic clinics. All children had a confirmed diagnosis of idiopathic toe-walking based on an exclusion of all other diagnoses. Children who ITW had not undergone any orthopaedic intervention (surgical or casting) 2 years prior to the study and had not received botulinum toxin-A injections in the 6 months prior to the study. Two children who ITW had received previous treatment. The remaining three children who ITW had significant fixed equinus contracture (Range 12–30° of plantarflexion with knee fully extended) and had received no orthopaedic intervention. Detailed participant characteristics are presented in Table 1. All TD children were free from neuromuscular and skeletal disorders and were free from lower limb injuries for 6 months prior to the study. This study was completed in accordance with both institutional and National Health Service (UK) Ethical Committee Approval (18/NW/0526). Written informed consent was obtained from parent/guardians and written assent given by children, in accordance with the Declaration of Helsinki.

**Table 1:** Detailed participant characteristics

Group	ID	Age (yrs.)	Height (m)	Mass (kg)	Contracture* (°)	Previous treatment
ITW	1	8	1.29	32.0	–	Insoles
	2	9	1.32	46.9	− 30	None
	3	6	1.29	23.9	–	Insoles and splints
	4	12	1.65	90.7	− 12	None
	5	6	1.36	32.7	− 25	None
TD	1	9	1.34	27.1	–	–
	2	12	1.45	42.8	–	–
	3	12	1.39	39.8	–	–
	4	7	1.20	26.7	–	–
	5	10	1.26	25.1	–	–
	6	11	1.43	36.7	–	–
	7	10	1.46	40.3	–	–
	8	6	1.26	23.3	–	–
	9	8	1.30	22.9	–	–
	10	13	1.39	46.3	–	–
	11	14	1.62	92.3	–	–
	12	9	1.40	39.5	–	–
	13	10	1.41	31.4	–	–
	14	11	1.48	34.6	–	–

*ITW* children who idiopathically toe-walk, *TD* typically developing children

*Contracture measured with knee fully extended

### Measurement protocol

Data were collected in one testing session at a university laboratory. Prior to data collection, a 5–10-min familiarisation period was given on a force-instrumented split-belt treadmill (Motek Medical, Amsterdam, The Netherlands) to ensure that (1) children could walk comfortably in their preferred gait pattern and (2) to identify self-selected walking speed.

Following familiarisation, anatomical features of the Achilles tendon, including the calcaneal insertion and Achilles tendon bend-point were identified through sagittal plane ultrasound images (Telemed Echoblaster, Vilnius, Lithuania) and marked on the skin, using a method previously reported (Harkness-Armstrong et al. 2020). Passive retro-reflective markers were positioned in accordance with a modified 6-degrees-of-freedom marker set. Modifications were that the calcaneal marker was placed directly onto the Achilles tendon insertion skin marker and an additional marker was placed distally to the Achilles tendon bend-point skin marker, to track the tendon path into the calcaneal insertion and to account for tendon curvature in the assessment of moment arm length. Measurements were obtained from the right leg of TD children, and the most affected leg in children who ITW, defined as the observed degree of plantarflexion during gait.

Participants walked barefoot in their preferred gait pattern, at a self-selected walking speed on the instrumented split-belt treadmill, whilst secured in an upper body fall-arrest harness for safety. Participants walked continuously until a minimum of 25 gait cycles were collected from the measured side. Three-dimensional (3D) kinetics from the treadmill and kinematics were collected using a 12-camera Vicon Vero system (Vicon, Oxford, UK) at sample rates of 1200 Hz and 120 Hz, respectively.

### Data processing

Static ultrasound images of the Achilles tendon insertion and bend-point were analysed manually in ImageJ software (ImageJ 1.51j8, USA), where the distance between the skin and mid-portion of the tendon was measured (Harkness-Armstrong et al. 2020). All kinematic and kinetic data were processed in Visual 3D software (C-Motion, Rockville, MD) using a custom-made pipeline. All data were low pass filtered with a cut-off frequency of 6 Hz. Stance phase was defined using a force plate threshold of 10 N. Data were then cropped to only include 20–90% of stance to (1) eliminate potential inaccuracies in centre of pressure calculations at low force outputs and (2) to only include positive (anterior) GRF moment arm values for TD children. All data were exported to Matlab (MathWorks R2019a, UK) for subsequent analysis, performed using a custom-made script.

**Fig. 1:**
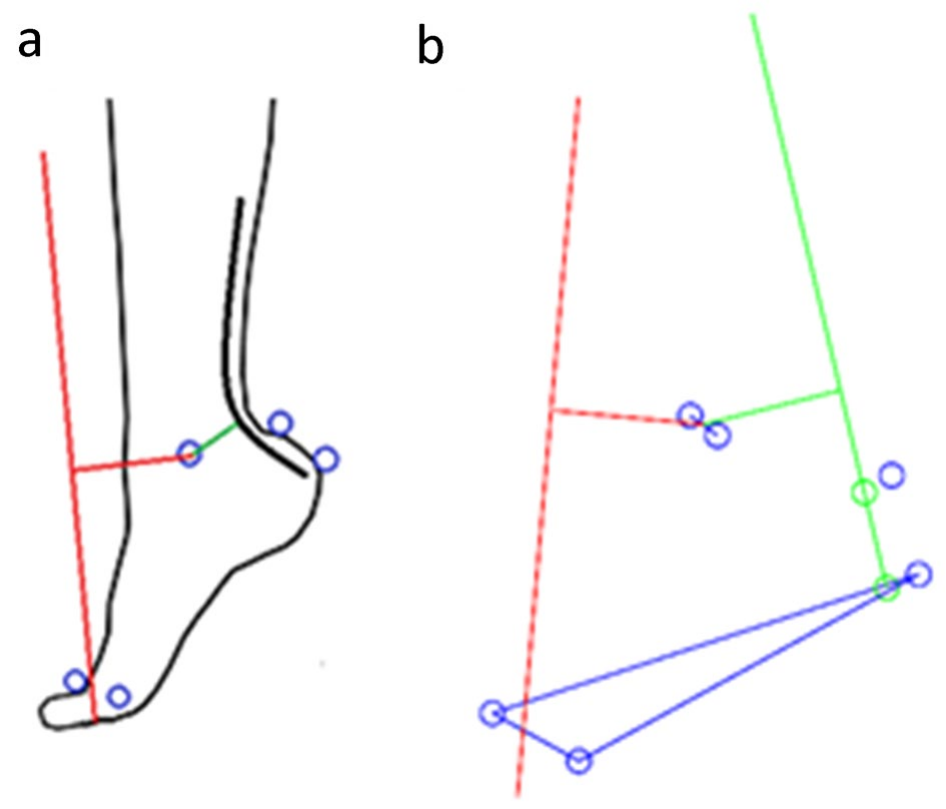
Visual representation of how the three-dimensional moment arms were calculated during gait. **a** 3D motion capture markers (blue circles) were positioned on the calcaneal insertion and bend-point of the Achilles tendon (Harkness-Armstrong et al. 2020), medial and lateral malleoli, and the 1st and 5th metatarsal heads. **b** Plotted 3D data of the captured physical motion capture markers (blue circles, as shown in Fig. 1a). The blue triangle outlines the foot plane and the transmalleolar axis is defined between the malleoli markers. Green circles represent the anterior-corrected motion capture markers along the foot plane (Harkness-Armstrong et al. 2020), through which the line of action of Achilles tendon force and the resultant moment arm (green lines) were plotted. Red lines represent the ground reaction force vector and the resultant moment arm

Achilles tendon and GRF moment arm lengths were calculated using a method previously reported (Harkness-Armstrong et al. 2020), which accounts for Achilles tendon curvature in extreme plantarflexed positions. In brief, the Achilles tendon path into the calcaneal insertion was tracked between the calcaneal and bend-point markers (Fig. 1a). Both markers were corrected anteriorly along the foot-plane by the respective distances measured from the static ultrasound images, to lie over the mid-portion of the tendon (Fig. 1b). Subsequent Achilles tendon and GRF moment arm lengths were normalised to height (normalised_height_) to control for between-participant variability in stature of children of different age and maturation stage. Height has been shown previously to be a predictor of Achilles tendon moment arm length in children (Kalkman et al. 2017) and young adults (Gallinger et al. 2020). All data were averaged for a minimum of 20 strides per participant.

Plantarflexor EMA was calculated throughout stance using Eq. (1):

1 $$EMA=\frac{{AT}_{MA}}{{GRF}_{MA}}$$

where, AT_MA_ is the Achilles tendon moment arm length, and GRF_MA_ is the GRF moment arm length.

To estimate the effect of EMA differences on the required muscle forces between groups, we approximated the plantarflexor muscle force requirements at peak plantarflexor moment using Eq. (2):

2 $${F}_{AT}= \frac{{GRF}_{v}}{EMA}$$

where, F_AT_ is the Achilles tendon force, and GRF_v_ is the vertical GRF normalised to body weight.

### Statistical analysis

All statistical analyses were completed in Matlab 2019a. All variables were checked for normal distribution. Achilles tendon moment arm length was the only variable to be normally distributed, therefore between-group comparisons were made using a Statistical Parametric Mapping (SPM1D) (Pataky et al. 2013) independent sample *t* test. Between-group comparisons of GRF moment arm length and plantarflexor EMA were therefore made using a Statistical Non-Parametric Mapping (SnPM1D) independent sample *t* test. Comparisons of plantarflexor EMA were made between 50 and 90% of stance only, as this is where meaningful plantarflexor moments are generated in both groups of children (≥ 0.5 Nm·kg^−1^). Significance was set at *p* < 0.05. Data are presented as means ± standard deviation (SD).

## Results

**Fig. 2:**
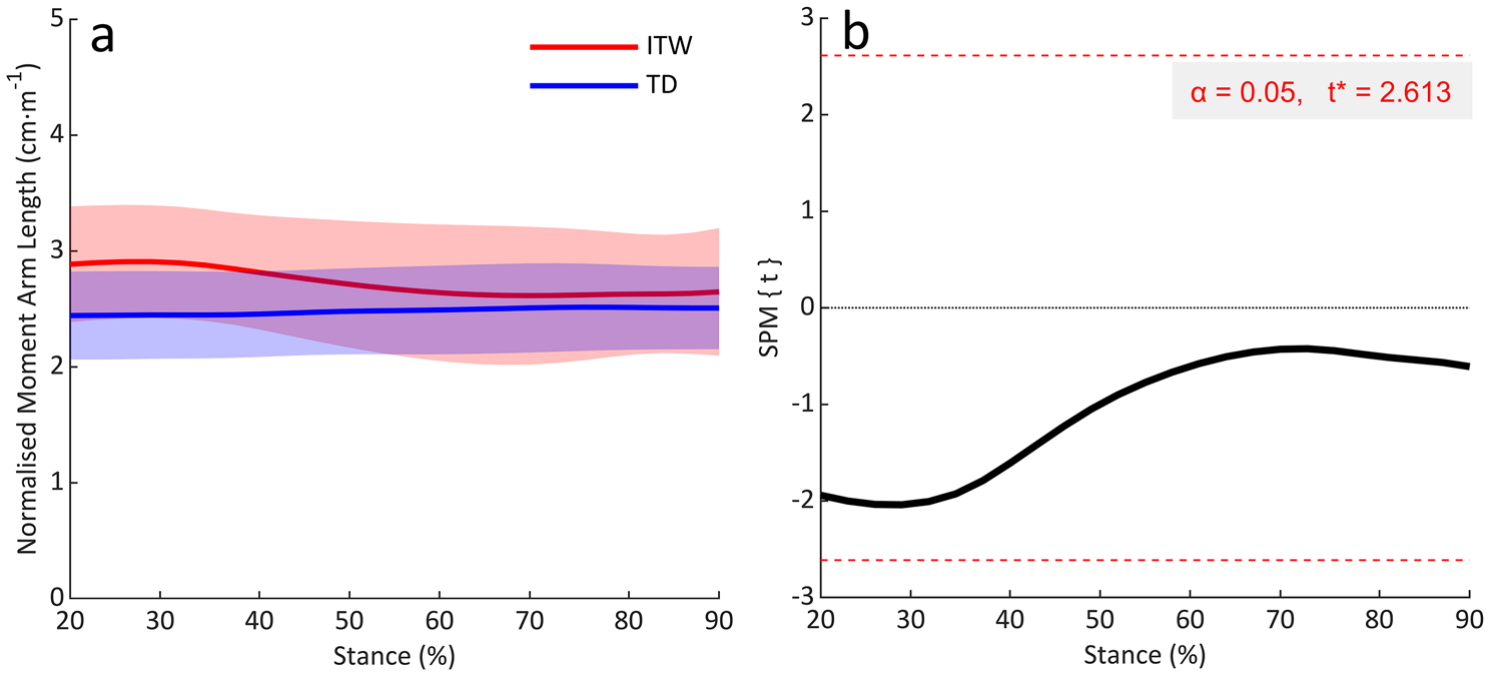
**a** Achilles tendon moment arm length normalised to height throughout 20–90% of stance. **b** SPM1D output for comparisons of normalised Achilles tendon moment arm length between groups. *ITW* children who idiopathically toe-walk, *TD* typically developing children

Gait kinematics and kinetics are presented in Supplemental Material. Self-selected walking speed did not differ between groups (0.76 ± 0.15 *vs* 0.86 ± 0.15 m·s^−1^; *p* = 0.186). Normalised_height_ Achilles tendon moment arm length remained relatively constant throughout stance in both groups (Fig. 2a) and did not significantly differ between children who ITW and TD children (mean across stance: 2.7 *vs* 2.5 cm·m^−1^; *p* > 0.05; Fig. 2b).

**Fig. 3:**
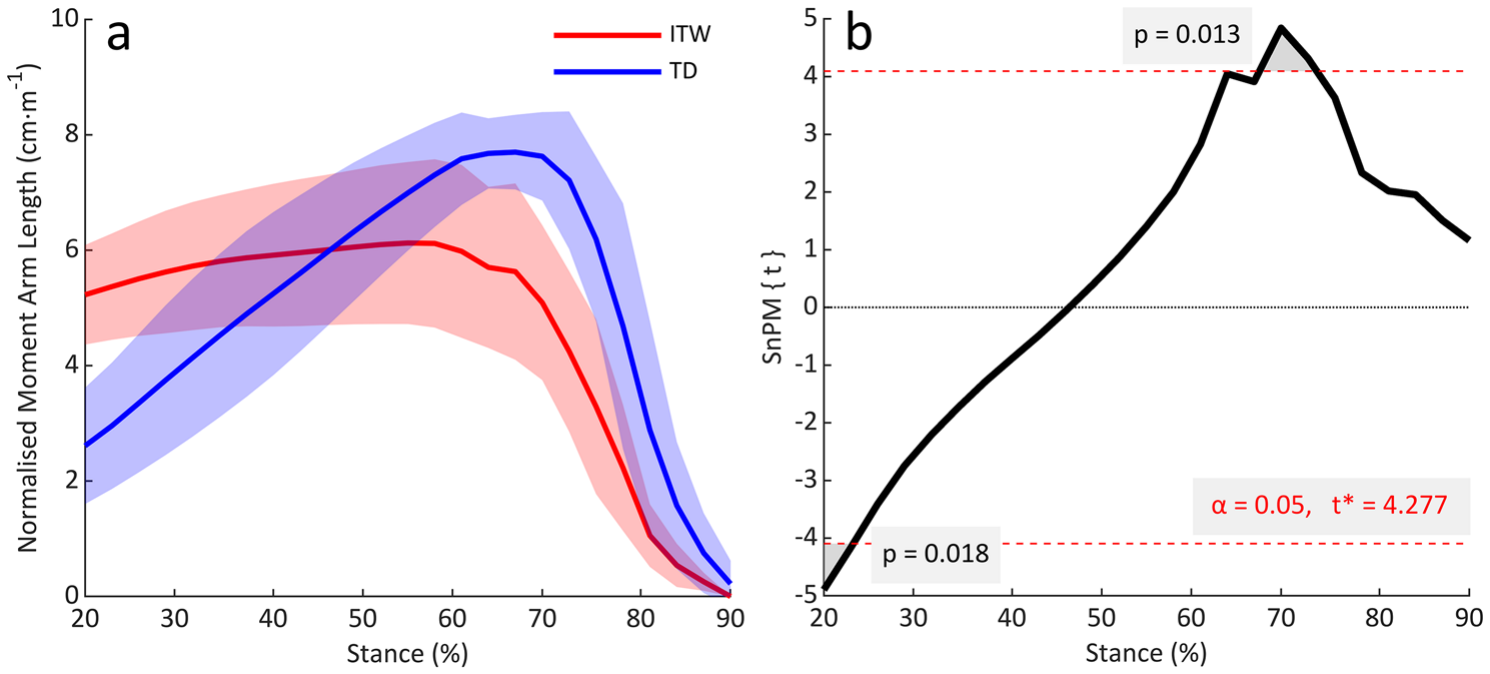
**a** GRF moment arm length normalised to height throughout 20–90% of stance. **b** SnPM1D output for comparisons of normalised GRF moment arm length between groups. *ITW* children who idiopathically toe-walk, *TD* typically developing children

Normalised_height_ GRF moment arm length increased until late stance for both groups. For TD children, this was from 2.6 to a peak of 7.7 cm·m^−1^ at 68% of stance. For children who ITW, this was from 5.2 to a peak 6.1 cm·m^−1^ at 58% of stance (Fig. 3a). This led to children who ITW having a significantly greater GRF moment arm length between 20–24% of stance (mean 5.3 *vs* 2.8 cm·m^−1^; %Δ = 89%; p = 0.001), and a significantly shorter GRF moment arm length between 68 and 74% of stance (mean 5.0 *vs* 7.5 cm·m^−1^; %Δ = 33%; *p* = 0.013) compared to TD children (Fig. 3b).

**Fig. 4:**
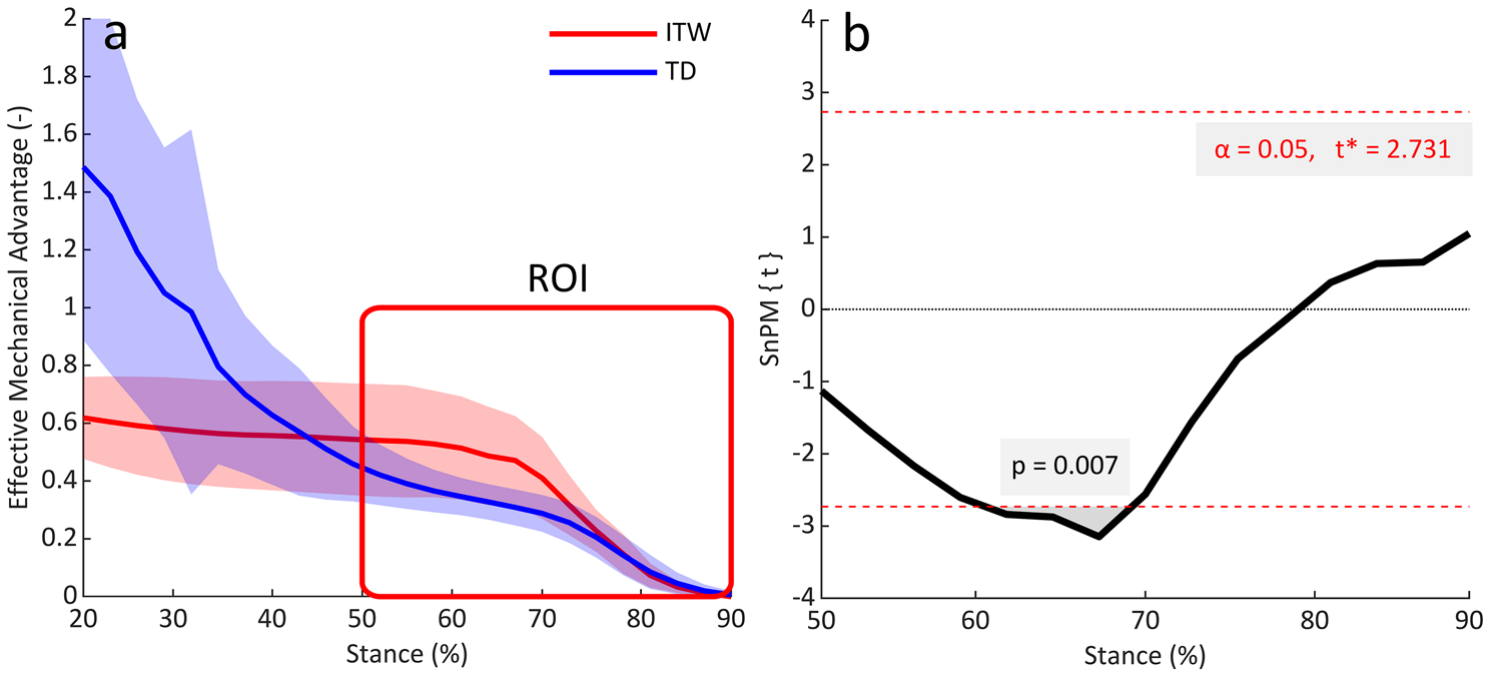
**a** Plantarflexor effective mechanical advantage throughout 20–90% of stance. Red box represents region of interest between 50 and 90% of stance, where meaningful plantarflexor moments (≥ 0.5Nm·kg^−1^) are generated in both groups. **b** SnPM1D output for comparisons of plantarflexor effective mechanical advantage from 50 to 90% of stance between groups. *ITW* children who idiopathically toe-walk, *TD* typically developing children, *ROI* region of interest

In the period when meaningful plantarflexor moments were generated in both groups (50–90% of stance), plantarflexor EMA was significantly greater in children who ITW than TD children (60–70% of stance; 0.5 *vs* 0.3; %Δ = 67%; *p* = 0.007; Fig. 4b). Consequently, the estimated required plantarflexor muscle force (normalised to body weight) at peak plantarflexion moment was 30% smaller in children who ITW (2.58 N) than TD children (3.67 N). Throughout stance, plantarflexor EMA of TD children decreased from 1.5 to 0.3 at peak plantarflexor moment (74% of stance). Whereas for children who ITW, there was only a slight decrease from 0.6 to 0.5 at peak plantarflexion moment (69% of stance) (Fig. 4a).

**Fig. 5:**
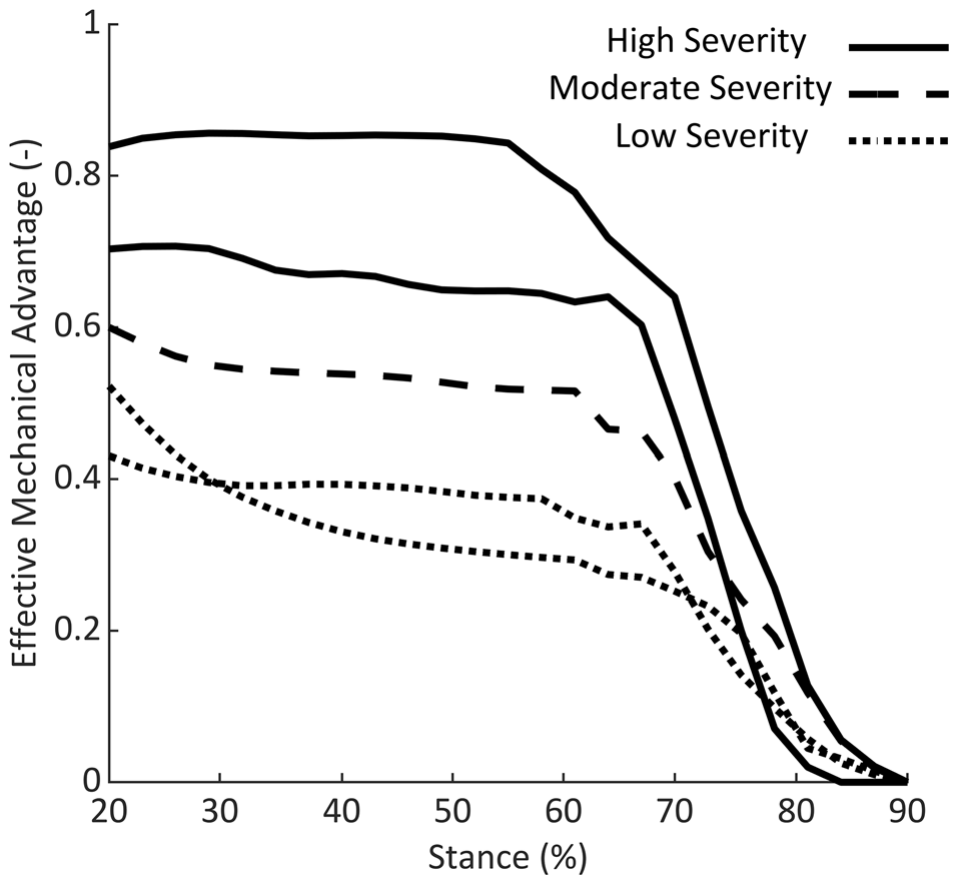
Individual plantarflexor effective mechanical advantage curves throughout 20–90% of stance for children who idiopathically toe-walk

It was notable that in the period of active plantarflexion (Region of Interest, Fig. 4a) the within-group variability of EMA was greater in children who ITW than TD children (0.2 *vs* 0.1). Examination of individual data (*n* = 5) showed that children who ITW with the highest severity of equinus (consistently more plantarflexed throughout stance) had the greatest EMA during propulsion (Fig. 5). Although statistical significance was not quite achieved (*p* = 0.059) when correlating plantarflexion angle and EMA at peak plantarflexor moment, a trend towards a high negative correlation (*r* = − 0.864) was observed.

## Discussion

This study is the first to quantify plantarflexor EMA in children who ITW. Children who ITW had, on average, a greater plantarflexor EMA than TD children in the period of stance where meaningful plantarflexion moments were generated in both groups. Consequently, the muscle force required for gait was estimated to be less in children who ITW. Therefore, clinical decision making should fully consider that interventions which restore a typical heel-to-toe gait pattern risk compromising this advantageous leverage and thus, may increase the plantarflexor muscle force requirements for gait.

Previous studies using two-dimensional sagittal plane models have shown that equinus gait decreases the GRF moment arm length, leading to a reduction in the plantarflexor muscle force requirements for gait (Hampton et al. 2003; Kerrigan et al. 2000). In the present study, we have confirmed that this mechanism applies in three-dimensional measurements. In TD children, the normalised_height_ GRF moment arm length increased throughout stance from 2.6 to a peak of 7.7 cm·m^−1^ (Fig. 3a), due to the natural progression of the GRF vector along the foot. This is consistent with previous work in typically developed adults (Giddings et al. 2000). However, in children who ITW, the normalised_height_ GRF moment arm was longer than typical at initial contact (5.2 mm) but only increased to a peak of 6.1 cm·m^−1^ (Fig. 3a), as the GRF vector did not progress along the foot in the same manner and remained more vertically orientated than for TD children. Consequently, comparable to previous work in other populations (Hampton et al. 2003; Kerrigan et al. 2000), equinus gait reduced the GRF moment arm length for children who ITW and thus, reduced the plantarflexor joint moment requirements for propulsion.

However, it has also been suggested that reducing the GRF moment arm length may be to compensate for anatomical changes in the Achilles tendon moment arm in children with CP (Kalkman et al. 2017). In this study, differences in both the absolute and normalised_height_ Achilles tendon moment arm length were not significant between children who ITW and TD children. However, the normalised_height_ Achilles tendon moment arm length was slightly larger in children who ITW. It is known that increased plantarflexion angle (Maganaris et al. 2000) and load (Maganaris et al. 1998) increase the Achilles tendon moment arm length. Therefore, between 20 and 50% of stance, where children who ITW were more plantarflexed and produced larger plantarflexion moments than TD children, this difference was more prominent (%Δ = 21%; Fig. 2a). However, between 50 and 90% of stance, where meaningful plantarflexion moments were generated in both groups, the difference in normalised_height_ Achilles tendon moment arm length was small (%Δ = 4%; Fig. 2a). Therefore, data from the present study suggests that the Achilles tendon moment arm lengths during gait are not functionally different between groups. Consequently, this may suggest that unlike children with CP (Kalkman et al. 2017), children who ITW do not have an anatomical change in the Achilles tendon moment arm, and toe-walking is likely not a compensation to achieve typical EMA. This interpretation is further supported by the fact EMA was larger in children who ITW and causes us to reject our hypothesis.

We compared plantarflexor EMA between 50 and 90% of stance, as this corresponded to the period of stance where meaningful plantarflexor moments were generated in both groups of children. The combination of a slightly greater Achilles tendon moment arm length with a significantly reduced GRF moment arm length led to a significantly (67%) greater plantarflexor EMA in children who ITW compared to TD children (60–70% of stance; Fig. 4a). Consequently, the required muscle force for any given propulsion force would be less for children who ITW. Indeed, the required plantarflexor muscle force at peak plantarflexion moment was 30% smaller in children who ITW than TD children. The EMA was also negatively associated with toe-walking severity for children who ITW. However, children who ITW are not weaker than TD children, and indeed operate close to their optimal joint angle during gait (Harkness-Armstrong et al. 2021). Therefore, it is unlikely that equinus is a strategy to reduce the muscle force requirements of gait for children who ITW, and rather, it appears more likely to be an additional consequence of the altered gait kinematics. Moreover, although a large EMA is beneficial when walking, it may be less favourable at higher speeds or when running (Ray and Takahashi 2020), and this should be investigated further with children who ITW to improve their participation.

All clinical interventions for children who ITW have a primary aim to restore dorsiflexion range of motion and a typical heel-toe gait pattern (Engström et al. 2013; Fox et al. 2006; Jahn et al. 2009; Satila et al. 2016). This will cause an increase in the GRF moment arm length during propulsion by restoring the natural progression of the GRF vector along the foot. Therefore, the plantarflexor EMA will inadvertently be compromised through clinical intervention. It is currently unknown what effect this will have on the required plantarflexor muscle force for gait, but data from this study indicate that if the gait kinematics matches those of TD children, the required muscle force could increase by up to 42% in children who ITW. Furthermore, clinical interventions may also, in the short-term, shift children who ITW away from their optimal joint angle (Harkness-Armstrong et al. 2021). Consequently, if recovery and post-intervention rehabilitation time is not sufficient to allow plastic changes in the muscle properties, or if a child does not have adequate muscle strength or endurance to meet the compound effects of reduced EMA and impaired utilisation of the force–length properties post-intervention, this may be a reason why current clinical interventions have poor medium to long term outcomes and high rates of recurrence (Dietz and Khunsree 2012; van Kuijk et al. 2014). Therefore, future work should quantify how plantarflexor EMA and thus, the muscle force requirements and movement economy are altered through clinical intervention. This will ensure that clinicians can decide on the appropriate treatment with full consideration of the implications, including considerations of foot pain (Fox et al. 2006) and gait stability (Soangra et al. 2021). Until now, it has not been feasible to quantify the EMA in children who walk in equinus. However, the simple and easy method demonstrated previously in adults (Harkness-Armstrong et al. 2020), and now in this study with children who ITW, could facilitate the implementation of such measurements into clinical practice.

Some limitations should be acknowledged. Although we took care in the placement of motion capture markers and in the acquisition of static ultrasound images, we cannot rule out that small errors may have been introduced by marker misplacement and skin movement artefact (Peters et al. 2010), or by alignment and pixelation errors (Goldstein 2000) impacting the correction of motion capture markers. However, this method has previously been shown to have good reliability (Harkness-Armstrong et al. 2020). We also used the trans-malleolar axis to define the ankle joint axis of rotation, whereas the true ankle axis operates around the talo-crural joint and varies in orientation across its range of motion (Barnett and Napier 1952; Lundberg et al. 1989). A functional joint calibration may have provided closer estimates to the true ankle joint axis of rotation (Wade et al. 2019), however this was not feasible for children who ITW. Finally, the sample size of children who ITW in this study may appear small, and this reflects a small population of children from which to recruit. Children who truly bilaterally ITW with no known or suspected cause/condition (CP, autism spectrum disorder, etc.), and whom have not undergone any recent clinical intervention (botulinum toxin-A in the 6 months prior and casting/surgery in the 2 years prior to the study) are few in number. Previous work has also suggested that there may be up to three different classifications of children who ITW (Alvarez 2007). Whilst our sample did include children with good variability in age, stature, mass and equinus severity, each classification of idiopathic toe-walking may not be “well” represented. Therefore, although we still detected statistically significant and clinically meaningful differences between the groups that are biomechanically sound, and thus can have confidence in these novel findings, confirmatory and long-term developmental studies using this method are required.

To conclude, we have shown that children who ITW do not present with alterations in the Achilles tendon moment arm length during gait. Thus, plantarflexor EMA is greater in children who ITW than TD children, due to a reduced GRF moment arm length. Consequently, children who ITW required 30% less estimated plantarflexor muscle force for propulsion. However, it is unlikely that equinus is a strategy to purposely reduce the muscle force requirements of gait, as underlying weakness does not present in these children who ITW. Further work should quantify the plantarflexor EMA pre and post clinical intervention, to assess alterations in this leverage and the resultant impact on muscle function.

## Supplementary Information

Below is the link to the electronic supplementary material.

Supplementary file1 (PDF 195 KB)

## Data Availability

The data that support the findings of this study are available from the corresponding author upon reasonable request.

## Abbreviations

CP: Cerebral palsy
EMA: Effective mechanical advantage
GRF: Ground reaction force
ITW: Idiopathically toe-walk
TD: Typically developing
