# Correction to: Children who idiopathically toe-walk have greater plantarflexor effective mechanical advantage compared to typically developing children

**DOI:** 10.1007/s00421-022-04950-2

**Published:** 2022-04-18

**Authors:** Carla Harkness-Armstrong, Constantinos Maganaris, Roger Walton, David M. Wright, Alfie Bass, Vasilios Baltzopoulos, Thomas D. O’Brien

**Affiliations:** 1 Research Centre for Musculoskeletal Science and Sports Medicine, Manchester Metropolitan University, Manchester, UK; 2 Research Institute for Sport and Exercise Sciences, Liverpool John Moores University, Tom Reilly Building, Byrom Street, Liverpool, L3 3AF UK; 3 Alder Hey Children’s NHS Foundation Trust, Liverpool, UK

## Correction to: European Journal of Applied Physiology 10.1007/s00421-022-04913-7

The original version of this article unfortunately contained a mistake. One of the author names is spelt incorrectly.

The correct author name should be Vasilios Baltzopoulos.

