# Supplementary material for: Children who idiopathically toe-walk have greater plantarflexor effective mechanical advantage compared to typically developing children
Source: Eur J Appl Physiol. 2022 Mar 16;122(6):1409–17. doi: 10.1007/s00421-022-04913-7 (PMC9132809; doi:10.1007/s00421-022-04913-7)
Supplement: Supplementary file 1 — Supplementary file1 (PDF 195 KB) [file 421_2022_4913_MOESM1_ESM.pdf]

Supplemental Material

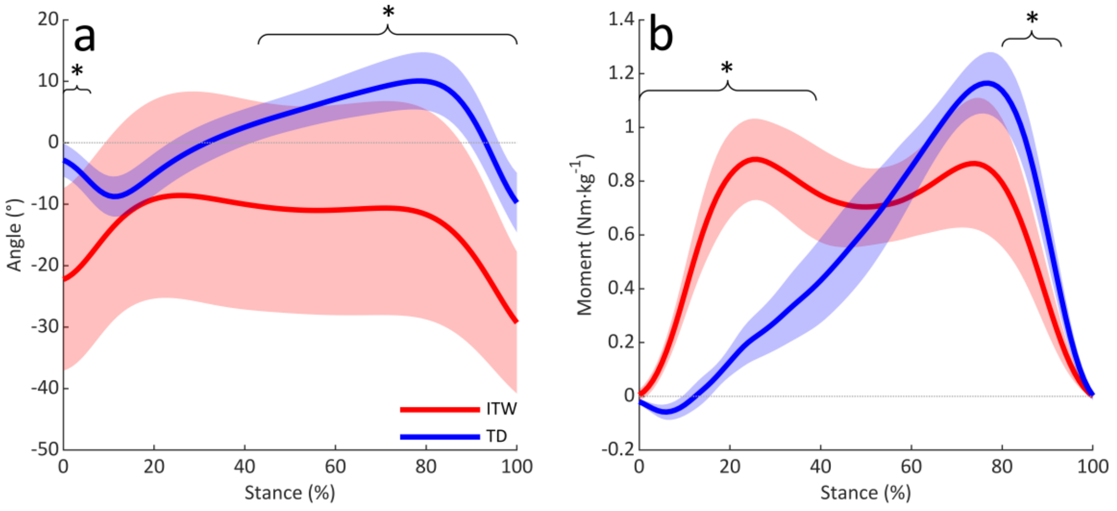

*Figure 1. (a) Ankle angle and (b) moment throughout stance of children who idiopathically toe-walk (red) and typically developing children (blue). *Significant difference between groups (p < 0.05).*
